# Investigating Bullying as a Predictor of Suicidality in a Clinical Sample of Adolescents with Autism Spectrum Disorder

**DOI:** 10.1002/aur.2292

**Published:** 2020-03-21

**Authors:** Rachel Holden, Joanne Mueller, John McGowan, Jyoti Sanyal, Maxim Kikoler, Emily Simonoff, Sumithra Velupillai, Johnny Downs

**Affiliations:** ^1^ NIHR South London and Maudsley NHS Foundation Trust Biomedical Research Centre London UK; ^2^ Canterbury Christ Church University Canterbury UK; ^3^ Department of Child and Adolescent Psychiatry, Institute of Psychiatry, Psychology and Neuroscience King's College London London UK; ^4^ Department of Psychological Medicine, Institute of Psychiatry, Psychology and Neuroscience King's College London London UK

**Keywords:** adolescents, clinical psychiatry, comorbid conditions, data‐driven techniques, epidemiology, longitudinal data analysis

## Abstract

For typically developing adolescents, being bullied is associated with increased risk of suicidality. Although adolescents with autism spectrum disorder (ASD) are at increased risk of both bullying and suicidality, there is very little research that examines the extent to which an experience of being bullied may increase suicidality within this specific population. To address this, we conducted a retrospective cohort study to investigate the longitudinal association between experiencing bullying and suicidality in a clinical population of 680 adolescents with ASD. Electronic health records of adolescents (13–17 years), using mental health services in South London, with a diagnosis of ASD were analyzed. Natural language processing was employed to identify mentions of bullying and suicidality in the free text fields of adolescents' clinical records. Cox regression analysis was employed to investigate the longitudinal relationship between bullying and suicidality outcomes. Reported experience of bullying in the first month of clinical contact was associated with an increased risk suicidality over the follow‐up period (hazard ratio = 1.82; 95% confidence interval = 1.28–2.59). In addition, female gender, psychosis, affective disorder diagnoses, and higher intellectual ability were all associated with suicidality at follow‐up. This study is the first to demonstrate the strength of longitudinal associations between bullying and suicidality in a clinical population of adolescents with ASD, using automated approaches to detect key life events within clinical records. Our findings provide support for identifying and dealing with bullying in schools, and for antibullying strategy's incorporation into wider suicide prevention programs for young people with ASD. ***Autism Res** 2020, 13*: 988‐997. © 2020 The Authors. *Autism Research published by International Society for Autism Research* published by Wiley Periodicals, Inc.

**Lay Summary:**

We investigated the relationship between bullying and suicidality in young people with autism spectrum disorder (ASD). We examined the clinical records of adolescents (aged 13–18 years old) with ASD in South London who were receiving treatment from Child and Adolescent Mental Health Services. We found that if they reported being bullied in the first month after they were first seen by mental health services, they were nearly twice as likely to go on to develop suicidal thoughts or behaviors.

## Introduction

Little is known about the association between bullying and suicidality in adolescent autism spectrum disorders (ASD). The importance of investigating the risk factors of suicidality, and in particular risks from bullying, is borne out by several important facts: (a) adults with ASD are more likely to end their lives by suicide compared with the general population [Hirvikoski et al., 2016; Kirby et al., 2019; Richards et al., 2019]; (b) young people with ASD are at 28 times higher risk of reporting suicidality compared with typically developing peers [Mayes, Gorman, Hillwig‐Garcia, & Syed, 2013]; (c) bullying is an established risk factor for the development of multiple mental health problems and suicidality in the general populations [Klomek et al., 2009]; and (d) bullying is known to be more prevalent in populations of adolescents with ASD [Mayes et al., 2013].

There are higher levels of suicide in people with ASD compared with the general population, and hence we need robust longitudinal studies that can investigate risk factors for suicidality on populations of young people with ASD. Potential causes, and the extent of their effects, need to be identified through longitudinal approaches. Only then will it be possible to prioritize which protective factors and risk factors should be targeted within prevention programmes. Currently the evidence base is limited: almost all studies conducted are cross‐section‐al [Hannon & Taylor, 2013; Hedley & Uljarević, 2018; Pelkonen & Marttunen, 2003; Zahid & Upthegrove, 2017] which do not provide clarity on cause and effect relationships. Methodological and sampling variations across the small number of relevant studies conducted have led to broad prevalence and risk estimates [Hebron & Humphrey, 2014; Zahid & Upthegrove, 2017]. For example, bullied groups with ASD have been shown to have large increases in risk of suicidality (>3 fold) relative to non‐ASD groups, but due to limitations in sample size these estimates have been imprecise and nonsignificant [Mayes et al., 2013]. Furthermore, most studies have only presented univariate analyses and have not taken account of potential confounders. Potential confounders include a wide range of biological, psychological, and social/demographic factors whose associations with suicidality have been identified. For example, depression and social isolation are both known to be associated with increased risk of suicidality in populations of adults with ASD [Hedley, Uljarević, Wilmot, Richdale, & Dissanayake, 2017]. This makes it difficult for clinicians or policy makers to understand the direct contribution of bullying to suicidality, and how to prioritize resources that mitigate its effects.

This current study aimed to address some of the main limitations of previous work and investigate the impact of bullying on suicidality. We used a historical cohort design within a clinical population of young people with ASD as an inception cohort. We measured baseline exposure of peer to peer bullying, and examined which young people developed suicidal outcomes over a 5‐year follow‐up period.

### Hypothesis

In a clinical sample of adolescents with ASD, bullying will be a significant predictor of incident suicidality after controlling for a number of potential sociodemographic and clinical confounders.

## Methods

### Sample and Setting

This study established a cohort of 1456 young people (aged 17 and under) with a diagnosis of ASD who had been first referred to South London and Maudsley NHS Foundation Trust (SLaM) between January 1, 2008, and December 31, 2013. This cohort was then followed up until June 1, 2016, or their 18th birthday. Data were extracted from Child and Adolescent Mental Health Service records via the Clinical Record Interactive Search (CRIS) [Downs et al., 2019; Perera et al., 2016]. At the time of the study, CRIS provided a pseudonymized database, containing both structured and free text data for over 35,000 young people referred to SLaM services. This includes proforma assessments (both risk and clinical), structured fields for recording medication, diagnosis and demographic information, and unstructured fields in which clinical correspondence and progress notes are included as free text.

Adolescents aged between 13 and 18 years who had face‐to‐face clinical meetings and a diagnosis of ASD recorded in CRIS were included in the study. ASD was categorized according to ICD‐10 diagnostic code (F84.0, F84.1, F84.5–9). Young people were included in the ASD group if they fulfilled ICD‐10 criteria for pervasive developmental disorder as this diagnosis is now encapsulated by an ASD diagnosis. The Autism Diagnostic Observation Schedule (ADOS) [Lord, Rutter, DiLavore, & Risi, 2000] was administered by experienced ADOS trained clinicians when the diagnosis was not clear. The final diagnosis was based on best clinical judgment considering all the available information [Volkmar et al., 2014] in child health clinics or Child and Adolescent Mental Health Services (CAMHS). Clinicians who make diagnoses of this kind have been trained specifically in differential diagnosis, including training on structured measures, such as the ADOS, ADI, and 3‐DI [Lord et al., 2000; Lord, Rutter, & Le Couteur, 1994; Skuse et al., 2004].

Adolescent ASD diagnoses were identified from the clinical record at any time point during the inception period (January 1, 2008, and December 31, 2013). Natural language processing (NLP) approaches were used to automatically filter free text and structured fields for ASD classification, as described in more detail in Downs et al., 2016; Downs, Lechler, et al., 2017. Validation of the accuracy of this approach to classify ASD diagnosis data extraction was carried out by a human clinical note review of 100 randomly selected cases from all CRIS CAMHS records. This data extraction method was found to have a high sensitivity (0.82) and PPV (0.86).

### Main Outcome

Incident suicidality was detected using a novel NLP application, fully described in Downs, Velupillai, et al. (2017). In brief, electronic health care records of the sample (e.g., their risk assessments, progress notes, correspondence) were screened by the NLP application and identified any mentions of suicidality related text (e.g., any information related to positive or negated mentions suicidality). All records identified as containing such mentions were then reviewed by two CAMHS clinicians (R.H., M.K.), who annotated the mentions as positive, negated, or unknown. A positive classification was defined as any mention in any record by the patient of suicidal ideation. This included passive thoughts of suicide (e.g., I wish I was dead) as well as active thoughts of suicide (e.g., I am going to jump off a bridge). Concerns about suicide risk expressed by caregivers were coded as positive for suicidality, as was treatment or risk management provided in response to suicide risk (e.g., hospitalization due to suicide risk). Self‐harming behaviors were coded as positive if subsequent assessed intent was established or if the young person clearly stated that they believed the act to be lethal. In addition, highly lethal behaviors (e.g., hanging) were classed as suicidality even if intent could not be clearly established within the extract. Unclear mentions of suicidality were classified after review by a consultant child and adolescent psychiatrist (J.D.). One hundred randomly selected documents from the sample were double annotated by the CAMHS clinicians. A document‐level assessment of interrater agreement for positive mentions of suicidality yielded an average Cohen's κ of 0.94 [Downs, Velupillai, et al., 2017]. A baseline period was defined as the 30 days from first face‐to‐face contact, and all time prior to first face‐to‐face contact. If suicidality was identified within this time period, these young people were excluded from the study, leaving an inception cohort of young people who were not clinically ascertained to be suicidal, this reduced the likelihood of reverse causation. This led to 314 young people being excluded from the study for this reason, 155 of these adolescents also reported bullying during the baseline period.

Adolescents were followed up until whichever came sooner from their 18th birthday, June 1, 2016, or reaching 5 years postbaseline—during which time some may have had continuous or intermittent periods under CAMHS care.

### Main Exposure

Exposure to bullying was detected within individual clinical records using Text Hunter (Version 3.0.6, Jackson et al., 2014), an NLP application that has been used to develop numerous applications within CRIS records [Downs et al., 2018; Fernandes et al., 2018; Jackson et al., 2017]. This application had several stages: (a) identification of prespecified keywords (e.g., bullied) within a database of documents; (b) provision of an interface for clinical reviewers to classify the keywords, using the context of the whole document, into positive, negative, or unknown classifications, forming a reference standard; (c) splitting the annotated dataset into a training and test set, then using a support vector machine learning approach to model the probability of the keywords as being positive; (d) selecting the threshold probabilities to classify a “positive” keyword; and (e) testing against the reference standard to provide an accuracy metric. Specifications for developing this bullying specific NLP application are included as supplementary material. In brief, a keyword terms list was generated from wildcard searches of documents containing $bull*. A CAMHS clinician (R.H.) annotated 200 documents containing bullying related terms into positive (evidence that the patient has been bullied, e.g., “their teacher is concerned that they are being bullied”), negative (evidence that the patient has not been bullied, e.g., “they reported getting on well with peers and never having been bullied”) or uncertain classifications (a mention of the term that neither confirms nor disconfirms bullying, e.g., “her twin sister has been bullied”). After the final version of the NLP application was applied to the sample, all positive outputs were reviewed. False positive classifications were identified and recoded, as well as positive mentions that denoted “bullying within the family setting.” This method led to a precision of 100% and a recall of 98% for bullying mentions in the final reference standard. For this study, bullying was regarded as an exposure when it was identified in the baseline period of observation, that is, 30 days from first face‐to‐face contact. As such, bullying as an exposure could not coincide with suicidality, as young people identified as suicidal in the baseline period were excluded from the sample. Extraction of bullying information was conducted independently and blind with respect to suicidality status.

### Covariates

Co‐occurring psychiatric disorders were diagnosed by CAMHS clinicians according to ICD‐10 criteria and extracted using methodology described in Downs et al. [2016]. Additional variables extracted from CRIS structured fields included sex, ethnicity, age at first recorded face‐to‐face clinical contact, Child Global Assessment Score (CGAS) [Shaffer, Gould, Brasic, Bird, & Aluwahlia, 1983], clinician rated risk of violence, risk of abuse, parental substance misuse, and parental mental health problems. These risk‐based items were coded as binary variables: no risk/low risk or moderate/high risk [Downs et al., 2016]. To be regarded as exposure variables, these risk factors had to be identified in the baseline observation period. Age at first face‐to‐face CAMHS contact was considered through adjustment in our multivariable analysis.

### Analysis Plan

All data analyses were performed in STATA version 14 [StataCorp, 2015]. Univariate analyses were conducted to detect sample differences at baseline between bullied and nonbullied groups. Independent *t*‐tests for continuous variables (e.g., age, CGAS score) and chi‐squared tests were used for sociodemographic and clinical categorical variables.

To examine the prospective association between baseline demographic, clinical exposures, and suicidality outcome, we used Cox regression to model the association between this baseline bullying and suicidality over a 5‐year follow‐up period from first presentation, or before the age of 18 years, whichever came first. After checking proportional hazards assumptions, the first analysis examined the unadjusted association of bullying on suicidality outcome. Subsequent adjustments were constructed by adding sequential blocks of potential individual‐level sociodemographic, co‐occurring psychiatric diagnoses and other clinical factors, and then family‐ and neighborhood‐level confounders. The final model examined adaptive function (CGAS score) as potential effect modifier between bullying and other the covariates on suicidality outcome. The survival analysis accounts for any potential variable follow‐up period between bullied and nonbullied groups. It represents the instantaneous positive suicide rate for an individual who has already survived to time *t*, the hazard ratio is an overall estimate of the ratio of the bullied and nonbullied hazards at equivalent points of time over the course of follow‐up.

### Ethics

In 2008, CRIS was approved by the Oxfordshire Research Ethics Committee C (reference 08/H0606/71+5) to perform secondary data analysis of pseudonymized clinical information searched for and retrieved from its database.

## Results

In total, 680 adolescents met the inclusion criteria for this study. Nearly a third of the overall sample reported bullying at baseline (201, 30%). The overall sample was mostly male (508, 75%) and of white ethnicity (377, 55%) with an average age at baseline (first contact) of 15 years. The average follow‐up time for the sample was approximately 2 years (mean days 700 [SD 447]); additional characteristics by suicidality outcome are displayed in Table 1.

**Table 1:** Clinical Sample Characteristics by Suicidality Outcomes

Baseline characteristics	Total *n* (% of overall sample)	Suicidality over follow‐up *n* (%)	No suicidality over follow‐up *n* (%)
Total sample	680 (100%)	128 (19%)	552 (81%)
Bullied	201 (30%)	56 (44%)	145 (26%)
Female	172 (25%)	48 (38%)	124 (22%)
Age (mean, SD)	15.2 (SD = 1.4)	14.8 (SD = 1.3)	15.3 (SD = 1.4)
White ethnicity	377 (55%)	78 (61%)	299 (54%)
Black	163 (24%)	29 (23%)	134 (24%)
Asian	37 (5%)	6 (5%)	31 (5%)
Mixed	12 (10%)	12 (9%)	56 (10%)
Other/not stated	35 (5%)	3 (2%)	32 (6%)
Neighborhood deprivation least deprived (1st tertile)	230 (36%)	52 (42%)	178 (34%)
2nd	210 (32%)	36 (29%)	174 (33%)
Most deprived (3rd tertile)	208 (32%)	37 (30%)	171 (33%)
Caregiver substance misuse	37 (5%)	8 (6%)	29 (5%)
Caregiver mental health disorder	108 (16%)	29 (23%)	79 (14%)
Risk of abuse (rated moderate or high)	150 (22%)	35 (34%)	115 (21%)
Risk of violence to others (rated moderate or high)	235 (35%)	44 (27%)	191 (35%)
Anxiety diagnosed	13 (2%)	4 (3%)	9 (2%)
Depression diagnosed	33 (5%)	21 (9%)	12 (4%)
Psychosis diagnosed	35 (5%)	13 (10%)	22 (4%)
ADHD	147 (22%)	34 (26%)	113 (20%)
ID	200 (29%)	20 (16%)	180 (33%)
SSRI prescribed	46 (7%)	13 (10%)	33 (6%)
Antipsychotic prescribed	102 (15%)	19 (15%)	83 (15%)
Mean CGAS score (SD)^a^	45.29 (15.7)	46.48 (13.2)	45.0 (16.3)
Mean duration of follow‐up in days (SD)	699.55 (447.2)	772.9 (446.32)	682.54 (446.10)

a Missing values *n* = 97.

In all, 128 adolescents (19%) had positive mentions of suicidality over the follow‐up period. In adolescents who developed suicidality over the follow‐up period, 56 (44%) of their records demonstrated bullying at the time of first clinical contact/assessment.

Table 2 displays characteristics of the sample by bullying exposure. Compared with the nonbullied sample, the bullied sample were more likely to be female (31% vs. 23%, *P* = 0.019) and significantly younger at baseline (14.8 years vs. 15.3 years, *P* < 0.001). The bullied sample also had a significantly larger proportion of recorded parent/carer substance misuse, moderate to high risk of abuse, and SSRI prescriptions. Intellectual disabilities and antipsychotic prescriptions were less prevalent in the bullied sample. Adolescents with recorded bullying had longer mean follow‐up periods (2.2 years vs. 1.8 years, *P* < 0.001) and higher mean CGAS scores (47.6 vs. 44.3, *P* < 0.001).

**Table 2:** Clinical Sample Characteristics by Bullying Status

Baseline characteristics	Total (%)	Bullied at baseline	Not bullied at baseline	Test statistics	*P* value
Total	680 (100%)	201 (30%)	479 (70%)		
Female	172 (25%)	63 (31%)	109 (23%)	*χ* ^2^ = 5.53	0.019
Mean age (SD)	15.2 (1.4)	14.8 (1.3)	15.3 (1.4)	*t* (678) = 4.30	<0.001
White ethnicity	377 (55%)	103 (51%)	274 (57%)	*χ* ^2^ (4) = 3.99	0.14
Black	163 (24%)	52 (26%)	111 (23%)		
Asian	37 (5%)	11 (5%)	26 (5%)		
Mixed	68 (10%)	25 (12%)	43 (9%)		
Other/not stated	35 (5%)	10 (4%)	25 (6%)		
Neighborhood deprivation least deprived (1st tertile)	230 (36%)	59 (31%)	171 (37%)	*χ* ^2^ (2) = 5.33	0.07
2nd	210 (32%)	72 (38%)	138 (30%)		
Most deprived (3rd tertile)	208 (32%)	60 (31%)	148 (32%)		
Caregiver substance misuse	37 (5%)	15 (7%)	22 (5%)	*χ* ^2^ (1) = 2.27	0.13
Caregiver mental health disorder	108 (16%)	40 (20%)	68 (14%)	*χ* ^2^ (2) = 3.45	0.06
Risk of abuse (rated moderate or high)	150 (22%)	69 (34%)	81 (17%)	*χ* ^2^ (1) = 24.98	<0.001
Risk of violence to others (rated moderate or high)	246 (36%)	82 (33%)	68 (16%)	*χ* ^2^ (1) = 28.49	<0.001
Anxiety diagnosed	13 (2%)	6 (3%)	7 (1%)	*χ* ^2^ (1) = 1.75	0.19
Depression diagnosed	33 (5%)	14 (7%)	19 (4%)	*χ* ^2^ (1) = 2.75	0.10
Psychosis diagnosed	35 (5%)	11 (5%)	24 (5%)	*χ* ^2^ = 0.06	0.80
ADHD	147 (22%)	35 (17%)	112 (23%)	*χ* ^2^ (1) = 2.98	0.084
Intellectual disability	200 (29%)	34 (17%)	166 (37%)	*χ* ^2^ (1) = 21.46	<0.001
SSRI prescribed	46 (7%)	20 (10%)	26 (5%)	*χ* ^2^ = 5.49	0.032
Antipsychotic prescribed	102 (15%)	18 (9%)	84 (18%)	*χ* ^2^ = 8.18	0.004
Mean CGAS score (SD)^a^	45.29 (15.7)	47.63 (13.2)	44.30 (16.6)	*t* (581) = −2.34	<0.001
Mean duration of follow‐up in days (SD)	699.6 (447)	792.8 (439)	660.4 (445)	*t* (678) = −3.55	<0.001

a Missing values *n* = 97.

Table 3 shows the full results of the regression analysis. Bullying was associated with a significantly higher risk suicidality over the course of follow‐up. Female gender, psychosis, and intellectual function in the normal range (i.e., absence of ID) were also associated with a higher likelihood of developing suicidality; after adjusting for neighborhood deprivation, affective disorder was also found to become a significant predictor. We found no significant association between bullying and suicidality after adjustment for CGAS scores, suggesting that adaptive function acts as an effect modifier between bullying and suicidality. However, even after adjustment for all other confounders, certain variables continued to significantly predict suicidality over the follow‐up period (CGAS scores, psychosis diagnosis, depression diagnosis, intellectual function in the normal range, and female sex).

**Table 3:** Cox Regression Models for the Association between Experience of Being Bullied and Later Suicidality in Adolescents with ASD (*n* = 680)

	Unadjusted			+ Adjusted for sociodemographic factors			+ Diagnostic and treatment factors			+ Risk and family characteristics			+ Neighborhood factors			+ Adaptive function (CGAS score)		
	Hazard ratio (HR)	95% CI	*P*	HR	CI	*P*	HR	CI	*P*	HR	CI	*P*	HR	CI	*P*	HR	CI	*P*
Bullied	1.82	1.28–2.59	0.001	1.80	1.26–2.57	0.001	1.57	1.12–2.32	0.010	1.58	1.09–2.28	0.015	1.59	1.09–2.30	0.016	1.33	0.88–2.00	0.19
Sociodemographic factors																		
Age				1.04	0.90–1.21	0.58	1.04	0.90–1.22	0.52	1.04	0.89–1.21	0.63	0.99	0.85–1.16	0.91	1.01	0.86–1.20	0.86
Female				1.94	1.35–2.79	0.001	1.99	1.38–2.86	<0.001	1.96	1.36–2.83	<0.001	1.97	1.36–2.87	<0.001	2.06	1.37–3.09	<0.001
Ethnicity		Ref																
White																		
Black				0.82	0.53–1.26	0.37	0.96	0.61–1.51	0.87	0.95	0.60–1.49	0.82	0.95	0.59–1.54	0.84	1.04	0.61–1.74	0.89
Asian				0.65	0.28–1.51	0.32	0.65	0.28–1.52	0.32	0.63	0.27–1.49	0.29	0.70	0.29–1.67	0.42	0.82	0.34–1.97	0.66
Mixed				0.82	0.44–1.52	0.53	0.85	0.46–1.59	0.62	0.82	0.43–1.53	0.53	0.79	0.42–1.50	0.48	0.90	0.15–1.56	0.75
Other/not stated				0.39	0.12–1.20	0.12	0.42	0.13–1.33	0.14	0.43	0.13–1.36	0.15	0.37	0.12–1.19	0.097	0.48	0.15–1.55	0.22
Clinical factors																		
Psychosis							2.32	1.24–4.35	0.008	2.37	1.26–4.46	0.007	2.46	1.30–4.67	0.006	2.98	1.46–6.08	0.003
Affective disorder							1.80	0.96–3.36	0.063	1.67	0.93–3.27	0.083	2.06	1.08–3.92	0.027	2.05	1.07–3.94	0.031
Anxiety disorder							1.31	0.48–3.60	0.60	1.26	0.46–3.48	0.66	1.32	0.48–3.66	0.53	1.62	0.58–4.58	0.36
Intellectual disability							0.39	0.24–0.65	<0.001	0.39	0.24–0.65	<0.001	0.39	0.23–0.65	<0.001	0.32	0.18–0.59	<0.001
ADHD							1.34	0.88–2.05	0.17	1.26	0.81–1.94	0.29	1.25	0.81–1.93	0.31	1.34	0.85–2.14	0.21
Antipsychotic medication							1.07	0.62–1.83	0.81	1.08	0.63–1.86	0.77	1.11	0.65–1.92	0.70	0.99	0.55–1.81	0.98
SSRI medication							1.19	0.64–2.19	0.58	1.22	0.65–2.28	0.53	1.10	0.58–2.08	0.77	0.85	0.42–1.73	0.66
Family factors																		
Risk of abuse										1.10	0.72–1.69	0.65	1.11	0.72–1.73	0.63	1.24	0.79–1.97	0.35
Risk of violence							Omitted due to collinearity											
Carer mental health disorder										1.35	0.85–2.13	0.21	1.22	0.83–2.10	0.24	1.17	0.71–1.93	0.35
Carer substance misuse disorder										0.65	0.29–1.44	0.27	0.67	0.30–1.50	0.36	0.68	0.30–1.55	0.36
Neighborhood factors																		
Increasing deprivation (tertiles)													0.83	0.65–1.05	0.12	0.85	0.66–1.10	0.22
Adaptive function																		
CGAS score																0.99	0.98–1.00	0.40

## Discussion

This study found, in a large clinical population of adolescents with ASD, that those who experienced bullying were nearly double the risk of later developing suicidal thoughts. The effect of bullying remained significant after accounting for a number of sociodemographic and clinical confounders. This finding is consistent with studies of populations of typically developing adolescents, which show bullying is strongly associated with suicidality [Holt et al., 2015]. This study is the first to use NLP techniques to identify an adverse life event, such as bullying, within routinely collected health records. Additionally, it is the first study to examine the longitudinal relationship between bullying and suicidality—an important adverse psychopathological outcome—in a clinical sample of adolescents with neurodevelopmental disorders.

Other findings of note include the near doubling of risk for suicidality among females with ASD relative to males. This is consistent with a fairly well‐established finding in general population‐based studies, which tend to identify female sex as a risk factor for suicidality in both adolescent and adult samples [Goldstein et al., 2012; Greenfield et al., 2008; Hawton, 2000; Hirvikoski et al., in press], and consistent with findings that females with ASD are at higher risk of death by suicide than men with ASD [Hirvikoski et al., 2016; Kirby et al., 2019]. There were no individuals within our cohort who died by suicide during the follow‐up period, but it is important to recognize that female sex in ASD emerged as a significant risk factor for suicidality within adolescence. More broadly, this finding adds further impetus to continuing to improve clinical recognition and support for adolescent females with ASD, who at present show increased vulnerability for suicidality and death by suicide relative to males.

Our study also provided some other important findings. We found those with dual diagnoses of ASD and psychosis at baseline were at significantly increased risk of developing suicidality. This is consistent with findings that ASD may be a complicating factor for adolescent recovery from psychosis [Downs, Lechler, et al., 2017; Sullivan, Rai, Golding, Zammit, & Steer, 2013]. Testing whether young people with ASD were at elevated risk of suicidality relative to non‐ASD adolescents with psychosis was beyond the scope of this study, but our finding highlights the need to maintain robust support and clinical treatment for young people with ASD following the onset of a psychotic episode.

We found significantly lower levels of reported bullying and suicidality in young people with ASD and ID compared to those with ASD and normal intellectual function. This is consistent with recent research by Hirvikoski et al. [in press], which found that individuals with ASD and ID were less likely to attempt suicide compared with individuals with ASD in the absence of ID. This runs against a prevailing view that developmental problems in childhood act cumulatively to predict further adversity [Kraemer, Lowe, & Kupfer, 2005]. Certainly within the general population, the prevalence of victimization/bullying in young people with ID is higher than in their typically developing peers, in those attending both special and mainstream schools [Knox & Conti‐Ramsden, 2010]. It may be the case that young people with ID and ASD are less likely to experience suicidality or bullying. However, we suspect that clinical interviews are not sufficiently sensitive screens in detecting adverse life events and suicidality in children with the combined social communication difficulties of ASD and ID, research suggests young people with ID are less likely to be assessed for risk of suicide [Dodd, Doherty, & Guerin, 2016]. Further research is needed to understand whether ID is truly a protective factor against the development of suicidality, or whether methodological issues causing an ascertainment bias of suicidality in youth with ID and ASD may be behind our findings.

### Implications

This research suggests that interventions aimed at preventing bullying in schools might reduce the risk of suicidality in young people with ASD that use CAMHS services. A recent study by Carrington et al. [2017] described the recommendations that young people with ASD and their caregivers made for bullying prevention and intervention. They found that adolescents and their parents recommended improved communication between school staff, parents, and pupils. For CAMHS clinicians, this research implies that asking about bullying is important and liaison with schools to manage problems with bullying could represent an important intervention.

Adolescents reported that they did not feel teachers were doing enough to prevent bullying. Evidence suggests that school‐based bullying interventions can reduce bullying [Vreeman & Carroll, 2007]. Vreeman and Carroll [2007] found that interventions work best when they are multidisciplinary. CAMHS clinicians, experienced in delivering systemic interventions, may be well placed to improve schools' communication and facilitate multidisciplinary working within schools. Ultimately, these school‐based interventions may reduce the degree of distress that young people with ASD presenting to CAMHS must overcome. ASD‐specific interventions that promote social inclusion could reduce the likelihood that young people with autism will experience bullying in schools. For example, better teacher training in understanding ASD, a more inclusive and tailored environment where all pupils feel safe and valued, and better pupil understanding of ASD, which improves integration may reduce the likelihood of bullying.

### Strengths and Limitations

A strength of this study was its use of electronic health records, which offer low‐cost access to large data sets. An advantage of this approach is that all records are included in studies and as a consequence possible selection bias is reduced. As the sample represents the whole clinical population of four South London boroughs it provides an ecologically valid picture. Using longitudinally collected clinical records avoids the response and recall bias that may arise in conventional survey research. Using a large sample size permitted the study to have sufficient statistical power to conduct analyses that remained robust after controlling for confounders. This addressed the sample size limitations of previous literature in the field. NLP offers an opportunity to extract quantitative data from the text‐based electronic health records of mental health patients. This includes all uploaded documents and all clinical notes. Using an NLP approach tailored to this population acknowledges the differences in presentation between adolescents with ASD and their typically developing peers. The suicidality and bullying extraction software application was originally validated by two clinicians with experience of working with children with ASD—it was not assumed that clinical terms used in typically developing children would generalize to ASD populations.

While using a large‐scale naturalistic cohort from electronic records offers tremendous potential for researchers to explore novel patterns using deep and complex datasets, there are drawbacks to this methodology. These data are not collected for the purposes of research and therefore clinical measurements are subject to the exigencies and habits of prevailing clinical practice. NLP approaches are not 100% accurate and the bullying and suicidality data will have contained both false positives and false negatives. The diagnostic shadowing of ASD over other clinical diagnosis is notable, particularly with anxiety disorders, which show a remarkably low prevalence (~2%) for a clinical sample, compared to reported rates in the general ASD population of young people [Simonoff et al., 2008]. Identifying co‐occurring psychiatric disorders, especially affective disorders, is important as targeting these for intervention could further reduce the risk of developing suicidality. As described by Horowitz et al. [2018] young people with ASD who talked about death, were more likely to have comorbid mood or anxiety disorders. Furthermore, the sample is drawn from an urban and suburban population, and although representing community CAMHS practice, the sample may not be generalizable to less urban populations. And finally, as with all the observational studies, the findings may be subject to residual confounding.

We carefully removed any cases of young people who had a prior report of suicidality at baseline. However, we permitted young people who be retained in the sample who received an autism diagnosis (or it was clinically recorded) after development of suicidality. We took the assumption that autism did not develop in adolescence and, therefore, the absence of earlier diagnosis did not mean ASD was not present from baseline. The exclusion of those with established bullying‐suicidality profiles reduced the risk of reverse causality and the effect of prior suicidality on later suicidality that may have been mediated by bullying. Our approach to isolate the effect of bullying from prior suicidality is a strength of the study, the approach using this approach may have led an underestimate of the impact of bullying on suicidality.

### Further Research

It was beyond the scope of this study to compare the outcomes of clinical populations of adolescents with ASD to the outcomes of other adolescents in contact with CAMHS. However, this study provides a clear rationale for doing so. It would be useful to understand whether adolescents with ASD are particularly vulnerable to certain risk factors as well as being potentially resilient to others. Furthermore, the developed bullying app did not assess whether bullying was historic or ongoing. Further research to assess whether interventions to stop bullying (i.e., bullying being absent at follow‐up) were effective in reducing suicidality would be a useful avenue for further research. In addition, research focusing on populations of adolescents with ASD from a nonclinical sample would be of use, as this population may not respond in the same way to experiences of bullying.

The degree and length of bullying experiences may be associated with the onset of suicidality. However, the scope of this paper was not to assess the type, duration, or severity of reported bullying and later suicidality, it was to establish whether “any report of bullying” was longitudinally associated with a risk of incident suicidality in a clinical population of adolescents with ASD. Further research focusing on the impact of different bullying typologies and severity on suicidality is warranted to enhance risk screening and the prioritization of treatment.

Self‐reported bullying rates should be an outcome of interest when assessing the effectiveness of social skills interventions. ASD‐specific interventions that promote social inclusion of autistic pupils need to be tested to see whether they impact the disproportionately high levels of bullying experienced by adolescents with ASD. Further research is needed to assess whether better teacher training and within class teaching on ASD could improve integration and reduce the likelihood of bullying.

## Conflict of Interest

No authors have any conflicts to declare.

## Supporting information


**Appendix S1**. Supplementary Material.
