# Supplementary material for: Investigating Bullying as a Predictor of Suicidality in a Clinical Sample of Adolescents with Autism Spectrum Disorder
Source: Autism Res. 2020 Mar 21;13(6):988–97. doi: 10.1002/aur.2292 (PMC8647922; doi:10.1002/aur.2292)
Supplement: Supplementary file 1 — Appendix S1. Supplementary Material. [file AUR-13-988-s001.docx]

**SUPPLEMENTARY MATERIAL**

**Summary of process to develop a ‘bullying’ mention extraction from electronic mental health records, using natural language tool TextHunter.**

100 human-rated documents (annotating positive, negative and unknown text instances according to the coding rules below) were provided as a training data to TextHunter and then tested whether annotations based upon the model corresponded with the annotations made to the training set. The first iteration of this application identified all positive instances of bullying that had been identified by the human annotator; however, it had a 28% false positive rate. In order to improve the accuracy of the application additional rules were created for surrounding words: “thought*bully”, “worry*bully” to automatically be coded as unknown. Large sections of standard text contained in a number of templates and forms which had been added onto electronic health records were also coded as unknown. A further 100 documents were annotated as a reference standard set. This improved the false positive rate to 14% leading to a Precision of 0.86 and Recall of 0.98. A further human-rater review of all positive mentions of bullying detected by the app across the entire sample (n=680) led to a precision of 0.99.

Bullying Application Specification

**Definition:**

Bullying is defined as unwanted aggressive behavior (verbal, social, cyber or physical) between school aged children outside of the family home (i.e. not between siblings). This application relies upon reported bullying. Young people or their families or schools will have reported involvement in bullying (either as bully, victim or bully/victim) to mental health services so that it forms part of their clinical record.

**Annotation rules:**

*Positive:*

Evidence of having been bullied

e.g.

“X has been bullied by a group of peers”

“In primary school X experienced extensive bullying”

*Negative:*

Evidence of not having experienced bullying

e.g.

“No bullying reported”

“X has not been involved in bullying”

*Unknown:*

Text is irrelevant and does not indicate a positive or a negative result

e.g.

“X’s twin is being bullied in school”

“He signed the school’s anti-bullying policy”

**Search terms:**

[space]bully*

Or

[space]bulli*

**Examples of excluded stock or template phrases:**

“Skills for life: 'Cyberbullying'”

“consequences of bullying behavior on the ward will be a deprivation of privileges

will be supported to make any formal complaint if being bullied according to the trust policy”

“staff are aware that bullying has been occurring on the unit and will remind young people of the unit rules”

“Bullying is defined as “

“No form of bullying of a service user from any source will be condoned by any staff “

“Examples of bullying behavior include”

“eliminate any of its service users from being bullied in line with its general” “safeguarding from abuse/harm policies and procedures”

“Bullying can also be a common experience for these youngsters who stand out from their peers. “

“AS Anti Bullying group”

“Coping with bullying”

“handling stressful situations including teasing/bullying”

“what is bullying?

Bully-Guard Body Armour'”

“bully body armour”
